# Solid state NMR assignments of a human λ-III immunoglobulin light chain amyloid fibril

**DOI:** 10.1007/s12104-020-09975-2

**Published:** 2020-09-18

**Authors:** Tejaswini Pradhan, Karthikeyan Annamalai, Riddhiman Sarkar, Ute Hegenbart, Stefan Schönland, Marcus Fändrich, Bernd Reif

**Affiliations:** 1 Helmholtz-Zentrum München (HMGU), Deutsches Forschungszentrum für Gesundheit Und Umwelt, Institute of Structural Biology, Ingolstädter Landstr. 1, 85764 Neuherberg, Germany; 2 Department of Chemistry, Munich Center for Integrated Protein Science (CIPS-M), Technische Universität München (TUM), Lichtenbergstr. 4, 85747 Garching, Germany; 3 Institute of Protein Biochemistry, Ulm University, Helmholtzstrasse 8/1, 89081 Ulm, Germany; 4 Medical Department V, Amyloidosis Center, Heidelberg University Hospital, 69120 Heidelberg, Germany

**Keywords:** AL amyloidosis, Variable light chain fibrils, Solid state NMR

## Abstract

The aggregation of antibody light chains is linked to systemic light chain (AL) amyloidosis, a disease where amyloid deposits frequently affect the heart and the kidney. We here investigate fibrils from the λ-III FOR005 light chain (LC), which is derived from an AL-patient with severe cardiac involvement. In FOR005, five residues are mutated with respect to its closest germline gene segment IGLV3-19 and IGLJ3. All mutations are located close to the complementarity determining regions (CDRs). The sequence segments responsible for the fibril formation are not yet known. We use fibrils extracted from the heart of this particular amyloidosis patient as seeds to prepare fibrils for solid-state NMR. We show that the seeds induce the formation of a specific fibril structure from the biochemically produced protein. We have assigned the fibril core region of the FOR005-derived fibrils and characterized the secondary structure propensity of the observed amino acids. As the primary structure of the aggregated patient protein is different for every AL patient, it is important to study, analyze and report a greater number of light chain sequences associated with AL amyloidosis.

## Biological context

Light chain (AL) amyloidosis is a lethal disease, caused by misfolding of immunoglobulin LC fibrils (Merlini et al. 2006). Circulating free LCs originate from monoclonal plasma cells that are affected by an underlying B-cell dyscrasia. LC amyloid deposits are found extracellularly in various critical organs of the body, especially affecting the heart and the kidney (Kyle et al. 1992; Baden et al. 2008). Currently, there is no cure for this disease, and the life expectancy rate after initial diagnosis is in order of 7 months (Wechalekar et al. 2013). Initially, the light chain protein is natively folded adopting an immunoglobulin fold in solution. It is believed that this globular protein undergoes a partial or a full unfolding reaction and populates oligomeric intermediate states before it finally forms fibrils that deposit in the inner organs (Souillac et al. 2003). Transient soluble, oligomeric states were found to be toxic and critical for initiation of amyloid fibril formation (Misra et al. 2019). In addition to a physical perturbation, also fibrils were shown to impair cardiomyocyte metabolism without causing significant cell death (McWilliams-Koeppen et al. 2015). The aggregation process and the molecular mechanism of misfolding of these LC proteins are so far not properly understood. Genetic variability via somatic hypermutation causes mutations in germline sequences leading to a large number of potentially amyloidogenic LCs fibrils. Aggregates in the AL amyloid patients contain primarily LC variable domain (V_L_), but also truncated forms of LCs and even full length LCs are found in the aggregates (Enqvist et al. 2009). The mechanism of cleavage is as well so far not understood.

We investigate here the fibrils formed from the V_L_ sequence of the LC of patient FOR005. The respective patient showed a dominant heart involvement (Annamalai et al. 2017). The protein sequence contains 109 residues and belongs to λ-III germline gene segment. In its native state, the protein is composed of primarily anti-parallel β-sheets and forms a dimer under crystallographic conditions. The primary structure of the λ-III LC FOR005 was obtained previously by cDNA sequencing (Annamalai et al. 2017). For reference, we determined the respective germline sequence (FOR005_GL), using the web tools abYsis (https://www.abysis.org/) and IMGT (https://www.imgt.org/). FOR005 and FOR005_GL differ in five amino acids in the variable germline segment, namely at residues S31Y, F48Y, R49G, S51N and A94G (from FOR005 patient to germline protein). In this study, we assign the rigid core and secondary structure elements of FOR005 fibrils using magic angle spinning (MAS) solid-state NMR spectroscopy.

## ﻿Methods and experiments

### Source of AL fibrils

AL amyloid fibrils were extracted from the heart of a patient suffering from advanced heart failure due to AL amyloidosis. The LC precursor of the AL protein sequence (FOR005) corresponds to “AL case 1” reported by Annamalai et al. (Annamalai et al. 2017). Fibrils employed for seeding are extracted from heart tissue as described there, and are referred to as ex vivo seeds.

### Protein expression and purification

Recombinant protein production were purified as described previously (Nokwe et al. 2016; Hora et al. 2017). Briefly, *E. coli* BL21 with a pET28(b +) vector containing the coding region for the FOR005 V_L_ domain was grown in minimal medium. ^13^C-glucose and ^15^N–NH_4_Cl were employed using a concentration of 2 g/L and 0.5 g/L, respectively. Expression was induced with 1 mM IPTG at an optical density (OD) of 0.6–0.8. After overnight expression at 37 °C, cells were harvested, and inclusion bodies were isolated. The dissolved protein from inclusion bodies was subjected to anion exchange chromatography followed by refolding using a 3.5 kDa dialysis tube and a buffer containing redox agents. Finally, pure protein was obtained using gel filtration chromatography. The total protein yield was on the order of 20–30 mg protein per liter of culture. To produce isotopically labelled protein, ^15^NH_4_Cl and ^13^C-glucose were employed as nitrogen and carbon sources, respectively.

### Transmission electron microscopy (TEM)

In order to confirm that fibrils have been formed, we performed TEM experiments. Formvar/Carbon 300 mesh copper coated carbon grids (Electron Microscopy Sciences) were exposed first to an argon atmosphere for 10 s. 5 μL of sample was then added to the grids and incubated for 1 min. Grids were subsequently washed with water and dried using filter paper. For staining, 10 μl of uranyl acetate (2%) was added for up to 30 s. Extra stain was removed from the grids using filter paper. Grids were visualized in TEM, employing an EM 10 CR or a LIBRA 120 plus (Zeiss, Germany) microscope.

### Fibril sample preparation for solid-state NMR

Fibrils were prepared using an initial protein concentration of 50 μM in 20 mM phosphate buffered saline (PBS), pH 6.5 at 37 °C. Protein solutions were incubated in a shaker (Thermo Scientific) at 120 rpm. 2.5–5% seeds were added to yield seeded fibrils. In addition, 0.05% sodium azide was used to prevent bacterial growth. Samples were incubated for 1 week to yield seeded fibrils. For all solid-state NMR samples, approx. 15 mg of protein have been employed. Protein aggregates were first centrifuged to reduce the volume to approx. 500 μL. Subsequently, the fibril slurry was sedimented for 1 h into a 3.2 mm thin wall ZrO_2_ MAS rotor (Bruker, Biospin), using a rotor filling tool (Giotto Biotech) and a L-100 XP ultracentrifuge (Beckman Coulter) equipped with an SW 32 Ti swinging bucket rotor operating at 28.000 rpm. The volume of the MAS rotor has been restricted to the active volume of the NMR coil using Teflon spacers.

### *In-vitro* prepared fibrils

To prepare *in-vitro seeds*, first non-seeded fibrils were grown. In all stages, a protein concentration of 50 μM (PBS buffer, pH 6.5 37 °C) have been used. These fibrils were subsequently sonicated for 3 min, and added to the purified, monomeric protein. The monomeric protein was filtered prior to the addition of seeds. This step was repeated two times. In all iterative steps, 5% w/v seeds were added to monomeric protein to finally select for the fastest growing polymorph.

### Solid state NMR spectroscopy

All solid-state NMR experiments were carried out at an external magnetic field of 17.6 T (corresponding to a ^1^H Larmor frequency of 750 MHz). 2D ^13^C,^13^C correlation experiments were acquired using either proton driven PDSD or DARR for mixing. Experiments involving aliphatic carbons were performed at a MAS frequency of 10 kHz, using a ^13^C,^13^C missing time of 50 ms. Experiments involving aromatic residues were performed at a MAS frequency of 16.5 kHz to avoid interference with rotation side bands. To assign the fibril NMR chemical shifts, conventional 3D NCACX and 3D NCOCX were recorded (McDermott et al. 2000; Pauli et al. 2001). For ^13^C,^15^N transfers, specific CP based RF building blocks were employed (Baldus et al. 1998). In addition, 3D CONCA and 3D CANCO experiments were performed to confirm and assign ambiguous residues (Li et al. 2007; Shi et al. 2009). In these experiments, optimal control CP (OC-CP) was used to increase sensitivity (Tošner et al. 2017, 2018). NUS spectra were reconstructed using the mdd algorithm (Orekhov et al. 2011). The assignment was done using the software ccpNmr 2.4.2.

### Reproducibility of fibril preparation using *ex-vivo* and *in-vitro* material for seeding

**Fig. 1:**
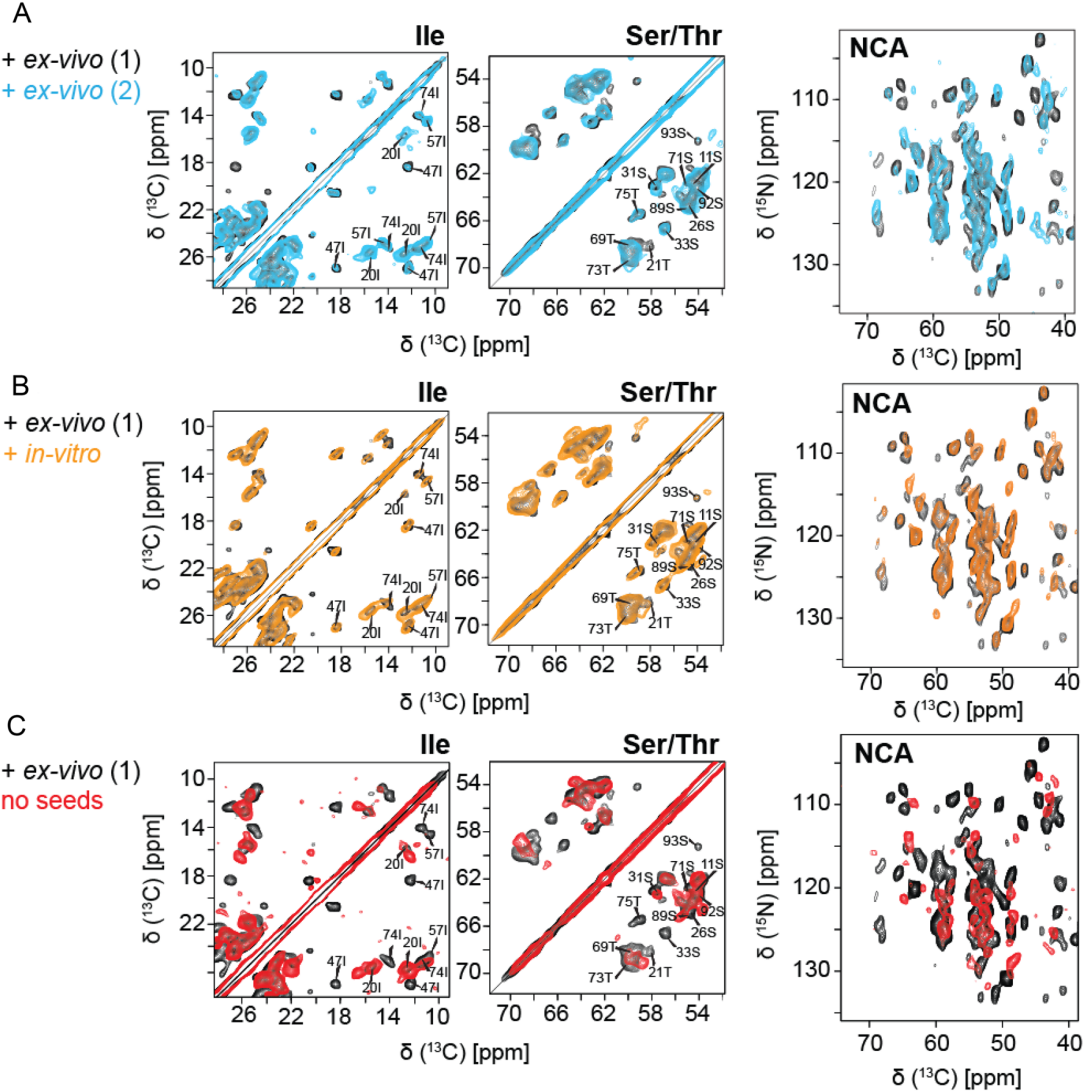
Reproducibility of the FOR005 fibril preparation and comparison of the seeding efficiency of ex-vivo and in-vitro seeds. **a** Superposition of 2D PDSD ^13^C,^13^C (left) and 2D ^15^N,^13^C correlation spectra (right) obtained for two patient FOR005 fibril preparations (black and cyan), using identical conditions to check the reproducibility of the preparation. The two preparations yield identical spectra in both experiments. **b** Superposition of 2D PDSD ^13^C,^13^C (left) and 2D ^15^N,^13^C correlation spectra (right) obtained for two patient FOR005 fibril preparations using ex-vivo seeds (black) and in-vitro (orange) prepared seeds. **c** Superposition of 2D PDSD ^13^C,^13^C (left) and 2D ^15^N,^13^C correlation spectra (right) obtained for FOR005 patient fibrils prepared with ex-vivo seeds (black) and prepared without seeds (red)

First of all, we wanted to test whether subsequent fibril preparations yield the same solid-state NMR spectra, and if sample preparation is reproducible. For this purpose, two fibril samples were prepared using recombinant FOR005 patient protein which was seeded in both cases with 5% *ex-vivo* fibrils. The resulting spectra were yielding very similar spectral patterns in both the 2D ^13^C,^13^C PDSD and the 2D NCACX experiment (Fig. 1a), suggesting that fibril structure is identical for these two samples. Similarly, we find that the patient protein FOR005 yields identical spectra whether fibrils are prepared using *ex-vivo* or *in-vitro* prepared seeds (Fig. 1b). The experimental cross peaks have similar linewidth and intensity in the two samples. The effectiveness of seeding is thus comparable for the two cases, suggesting that the seeding effects of the *ex-vivo* or *in-vitro* aggregates are highly similar. By contrast, seeded and non-seeded FOR005 patient fibril spectra (Fig. 1c) show very different chemical shift patterns in both the 2D PDSD and 2D NCACX experiment, indicating that the non-seeded fibrils have a differing structure. In all preparations described above, the same experimental conditions have been employed.

### Assignment and data deposition

**Fig. 2:**
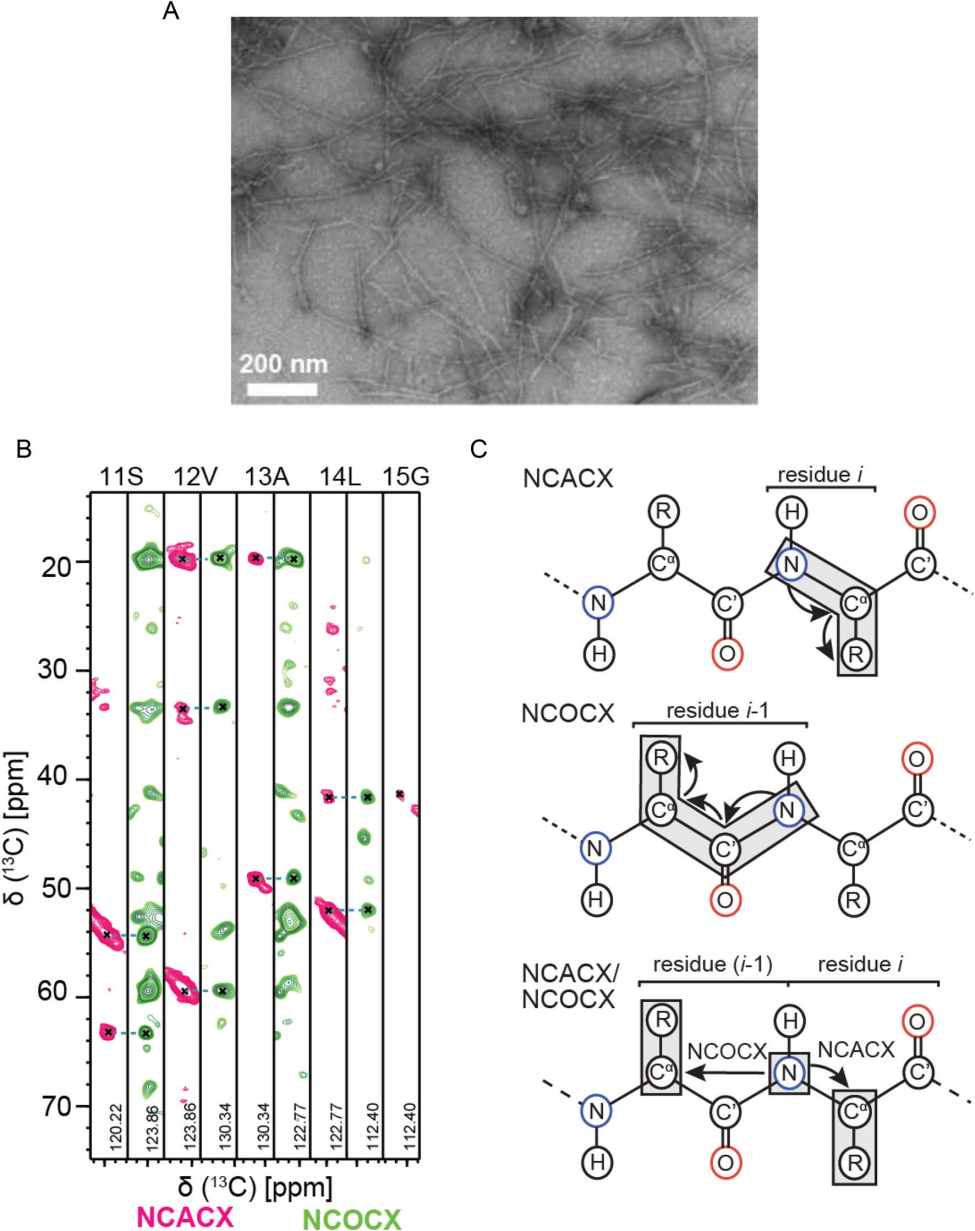
Solid-state NMR resonance assignments of patient FOR005 AL fibrils. **a** TEM image of FOR005 patient fibrils employed for solid-state NMR experiments. **b** 2D strip plots extracted from the 3D NCACX and 3D NCOCX experiments focusing on residues S11-G15. **c** Assignment strategy using 3D NCACX and 3D NCOCX experiments

In order identify the fibril core, we recorded 3D MAS solid state NMR spectra using uniformly labeled FOR005 fibrils. To ensure a high quality of the spectra, we have grown labeled fibrils using *ex-vivo* seeds. Seeds are extracted from heart tissue as described previously (Annamalai et al. 2017). The fibrils prepared for NMR this way show a high degree of structural homogeneity as judged by TEM (Fig. 2a).

Sequential assignment was obtained using standard 3D NCACX and 3D NCOCX experiments (McDermott et al. 2000; Pauli et al. 2001), together with 3D CONCA and 3D CANCO experiments (Li et al. 2007; Shi et al. 2009) to yield complimentary assignment information. Examples for 2D strips extracted from the 3D NCACX and 3D NCOCX are represented in Fig. 2b. The assignment strategy is shown in Fig. 2c.

By MAS solid-state NMR, we observe only one set of resonances. This observation is in contrast to an earlier study by Annamalai et al. who have shown, for a different set of conditions, that *ex-vivo* and *in-vitro* fibrils have different morphologies (Annamalai et al. 2017). Molecular dynamics simulations and solid-state NMR experiments have shown that the energetics for straight and twisted fibrils are rather similar, while at the same time the peptide structure is preserved (Jimenez et al. 2002; Matthes et al. 2014; Periole et al. 2018). This might explain that differences in fibril morphology by negative stain electron microscopy must not necessarily be related to large conformational changes in the protein backbone.

**Fig. 3:**
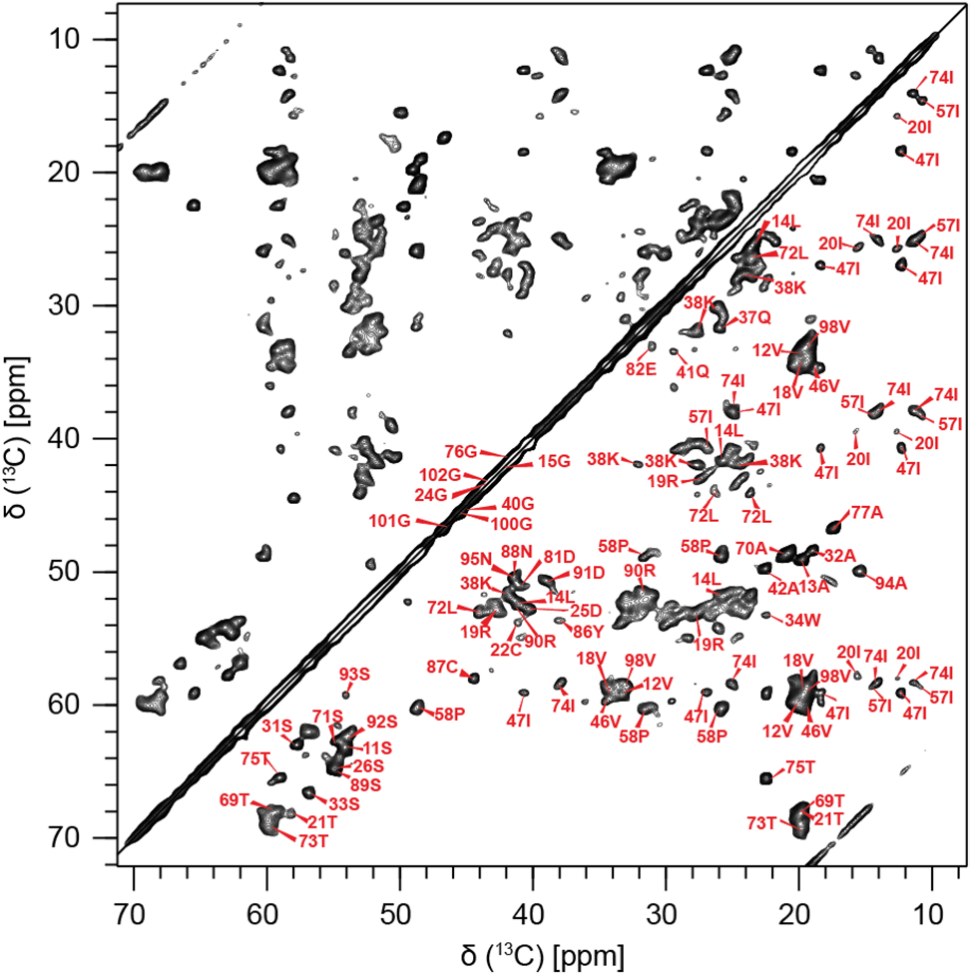
Assignments of AL FOR005 fibrils. 2D ^13^C,^13^C PDSD correlation spectrum of the aliphatic region of the spectrum of FOR005 fibrils with assignments. The spectrum was recorded using a MAS rotation frequency of 10 kHz and at an external magnetic field of 17.63 T, corresponding to a ^1^H Larmor frequency of 750 MHz

In total, we could observe 68 out of 109 spin systems. 55 out of the 68 spin systems have been unambiguously sequentially assigned. The sequential assignment of the resonances of the fibril sample is thus 80% complete with respect to all observable peaks. Figure 3 shows a 2D ^13^C,^13^C proton driven spin diffusion (PDSD) for FOR005 patient fibrils seeded with *ex-vivo* material, together with assignments. The focus is put on the aliphatic region of the spectrum. The ^13^C line widths are on the order of 90 Hz, and show no sign of structural polymorphism. For 13 spin systems, no sequential connectivities could be obtained, presumably due to the low sensitivity in the respective 3D experiments. However, for these spin systems the amino acid type could be identified. Assigned spin systems are indicated in the figure.

2D dipolar assisted rotational resonance (DARR) (Takegoshi et al. 2001) experiments, as well as 2D PDSD experiments performed at a MAS frequency of 16.5 kHz was yielding the assignment of aromatic residues. Among the aromatics, one tryptophan at position 35 is clearly visible and assigned in the 2D ^13^C,^13^C PDSD and DARR experiments, setting the MAS spinning frequencies to a value of 20 kHz. We also observe Try and Phe spin systems. Out of the three 3 phenylalanine residues, F99 is sequentially assigned. Cys-22 and Cys-87 could be sequentially assigned. Their chemical shifts are typical for cysteines in the oxidized state. This finding is in agreement with an intact disulfide bond in both the two recent cryo-EM structures and the chemical shifts of 6aJL2_R25G fibrils (Lecoq et al. 2019; Radamaker et al. 2019; Swuec et al. 2019).

**Fig. 4:**
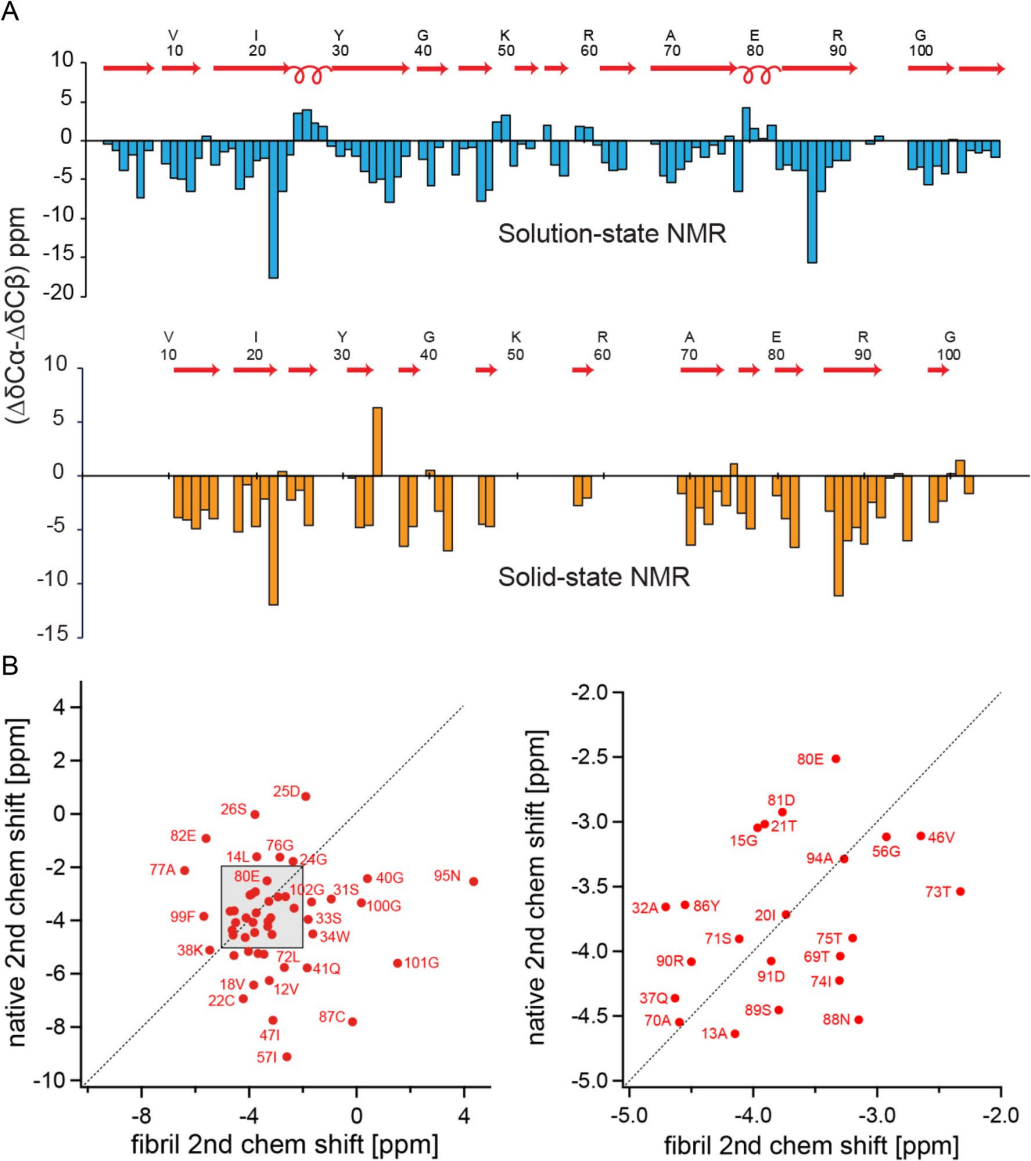
Comparison of solution- and solid-state NMR chemical shifts for FOR005.** a** Secondary NMR chemical shifts for monomeric, natively folded FOR005 in solution (top, light blue), and in the fibril state (bottom, orange). In each plot, the differences between chemical shifts of Cα and Cβ with respect to their random-coil chemical shifts are shown. **b** Cα secondary chemical shift correlation plot for folded FOR005 protein in solution (vertical axis) and FOR005 fibrils (horizontal axis). The figure on the right shows the shaded region of the left-hand figure enlarged. The cross-correlation coefficient R is on the order of R=0.015, indicating that the immunoglobulin-like fold of the native protein is not conserved in the fibril structure

Recently, domain swapping has been suggested as a mechanism to explain immunoglobulin light chain deposition (Bennett et al. 2006; Sonnen et al. 2010). In addition to the amyloid fibrils, we analyzed solution-state NMR data of the monomeric protein in buffer to yield NMR chemical shifts of the native state. If the conformation of FOR005 in the fibril state would resemble the conformation in the native structure, the secondary chemical shifts in the solid-state and in solution should be rather similar. We find, however, that secondary chemical shifts of the fibril and of the native monomer are very different (Fig. 4), suggesting that swapping of β-strands can be ruled out to yield a conversion from the natively folded monomeric protein to the fibril state.

Taken together, we have reported here the chemical shift assignments and the identification of the amyloidogenic core of the patient derived antibody light chain protein FOR005. At the moment, work is going on in the laboratory to determine the topology of the FOR005 fibril structure using MAS solid-state NMR.

## Data Availability

Solution-state and MAS solid-state NMR chemical shift assignments for native FOR005 and FOR005 V_L_ fibrils can be accessed on the BioMagResBank (BMRB) under entry number 50211 and 50192, respectively.
